# Relationships Between Reaction Time, Selective Attention, Physical Activity, and Physical Fitness in Children

**DOI:** 10.3389/fpsyg.2019.02278

**Published:** 2019-10-15

**Authors:** Rafael E. Reigal, Silvia Barrero, Ignacio Martín, Verónica Morales-Sánchez, Rocío Juárez-Ruiz de Mier, Antonio Hernández-Mendo

**Affiliations:** ^1^University of Málaga, Málaga, Spain; ^2^Department of Methodology for Behavioral Science, University of Granada, Granada, Spain; ^3^Department of Social Psychology, Social Work, Anthropology and East Asian Studies, University of Málaga, Málaga, Spain; ^4^Department of Evolutionary Psychology and Education, University of Málaga, Málaga, Spain

**Keywords:** reaction time, selective attention, FITLIGHT trainer, physical activity, physical fitness

## Abstract

The objective of this study was to analyze the relationships between simple and complex reaction times (RTs) with the physical activity performed weekly, the physical fitness and selective attention in children of the third cycle of primary education. Participants were 119 children aged between 10 and 12 years (*M* = 10.71; *SD* = 0.77). The instruments used for data collection were the D2 attention test to analyze selective attention, various tests of the Eurofit and ALPHA-Fitness Battery to evaluate the physical condition, a bioimpedanciometer Tanita TBF 300 to evaluate the body composition, and the FITLIGHT Trainer to measure the simple and complex RTs. The group that carried out more weekly physical activity used less time in simple (*p* < 0.05, *d* = −0.68, 95% CI [−1.19, −0.17]) and complex RT tests (*p* < 0.05, *d* = −0.63, 95% CI [−1.14, −0.12]). Also, the results showed that the simple RT was related in a significant way with physical fitness, while the complex RT was related significantly to attentional capacity and physical fitness.

## Introduction

Reaction time (RT) is a relevant variable in areas such as sports, academics, and other tasks of daily life (Metin et al., 2016; Sant’Ana et al., 2016). It can be defined as the time that elapses from when a stimulus appears until a response is given and is considered a good measure to assess the capacity of the cognitive system to process information (Jensen, 2006; Kuang, 2017). From a physiological point of view, RT is a complex phenomenon whose functioning has been studied by numerous researchers (Kuang, 2017). The RT depends on the speed of the sensorimotor cycle, composed by the detection of the initial stimulus, transfer of the information through the afferent nerves, generation of the response from the central nervous system, and final response (Adleman et al., 2016; Greenhouse et al., 2017).

There are differences between simple and complex RTs. The first is defined as the interval time between when a stimulus appears, its detection, and the given response (Jayaswal, 2016). The second involves the identification and selection of a response to various stimuli (Boisgontier et al., 2014). The simple RT is significantly shorter than the complex RT (Vences de Brito et al., 2011). The factors that influence the RT are numerous, being able to differentiate between those dependent on the own person and those related to the stimulus (Baayen and Milin, 2010). Among the first can be included the fatigue, physical condition, experience, motivation, gender, age, or dominance of the body member with which one responds. Second, the physical characteristics of the stimulus, its intensity, or duration (Der and Deary, 2006; Woods et al., 2015; Jayaswal, 2016).

In the set of internal factors, the influence of cognitive processes has been described as elements that determine the RT (Deary and Der, 2005; Leckie et al., 2014). Among them, attention would be a variable involved in the RT manifested by a person, which has been suggested in previous research (Prinzmetal et al., 2005; Vaportzis et al., 2013; Jehu et al., 2015). Attention is a cognitive function involved in the activation and selection processes, distribution, and maintenance of psychological activity (Chun et al., 2011; Greimel et al., 2011). It is a process of great anatomical and functional complexity, being able to differentiate manifestations as arousal, focal, selective, divided, alternating, or sustained attention (Petersen and Posner, 2012; Tamm et al., 2013). Specifically, selective attention would allude to the ability to attend to some specific stimuli and ignore others (Giuliano et al., 2014; Gomez-Ramirez et al., 2016).

It has been highlighted that physical activity and sports would be related to improvement in RT (Jain et al., 2015; Okubo et al., 2017; van de Water et al., 2017; Walton et al., 2018). The RT can be deliberately trained (Rabiner et al., 2010; Kirk et al., 2017), and physical activity and sports allow development of a wide variety of actions that would influence its development (Lynall et al., 2018; Walton et al., 2018). It is relevant in individual sports such as swimming or athletics, because it is necessary to respond quickly to start a movement (Nuri et al., 2013; Tønnessen et al., 2013). In other adversary or collective sports, such as badminton, karate, football, or basketball, RT is essential in multiple game situations, because athletes need to make quick decisions to be successful in their actions (Ruschel et al., 2011; Mudric et al., 2015; van de Water et al., 2017). Some studies had also shown that more fit people would be associated with less RT in a set of tasks (Luque-Casado et al., 2016; Westfall et al., 2018).

Previous research had also shown that physical activity and exercise and the improvement of physical fitness could support the development of cognitive functioning and specifically different aspects of attention (Hillman et al., 2009; Kao et al., 2017; Reloba-Martínez et al., 2017). For this reason, it could be considered that the practice of physical exercise and the development of physical condition could have an impact on RT, directly by the training of the capacity to respond to a given stimulus and indirectly by the impact it would have on cognitive functioning (Gentier et al., 2013; Syväoja et al., 2014).

To evaluate the RT, there are instruments, such as the FITLIGHT Trainer^TM^ (FITLIGHT Sports Corp., Canada) or the Dynavision^TM^ D2 Visuomotor Training Device (Dynavision International LLC, West Chester, OH), that have been implemented in different studies (Appelbaum and Erickson, 2018). Specifically, with the FITLIGHT Trainer, several investigations have been carried out. For example, Zwierko et al. (2014) conducted research with the FITLIGHT Trainer system, which showed that non-athletes had longer RTs than athletes. Likewise, Fischer et al. (2015) used this instrument for the training and analysis of the RT in the United States Air Force. On the other hand, Zurek et al. (2015) investigated the simple and complex RTs of 10 football players who had undergone knee surgery and a rehabilitation program to assess their recovery.

The literature consulted has highlighted the relationships between RT and variables such as selective attention and physical condition, although there are no previous studies that analyze them together in the preadolescent population. Therefore, the objective of the present study was to analyze the relationships between RT, selective attention, concentration, and physical condition in a sample aged from 10 to 12 years.

## Materials and Methods

### Participants

One hundred nineteen students (65 boys and 54 girls) participated in the study, aged between 10 and 12 years old (*M* ± *DT*: age = 10.71 ± 0.77 years; height = 1.45 ± 8.21 cm; weight = 42.58 ± 9.87 kg; body mass index = 19.96 ± 3.27 kg/m^2^; fat mass = 22.73 ± 8.37%) from Alcalá la Real (Jaén, Spain). All of them were in the fifth and sixth years of primary school and did not present any physical or psychological difficulties that could affect the study.

### Measures and Instruments

#### Reaction Time

The FITLIGHT Trainer (FITLIGHT Sports Corp., Ontario, Canada) was used to measure the RT. This is a wireless system consisting of eight sensors, which were placed on a table 1 m high and drawing a semicircle. They had a separation between them of 20 cm, with 40 cm from the central point. To perform the task, the student stood in front of it with his hand in contact with the table. Two tests were performed (simple RT and complex RT). The simple reaction test (SRT) was performed with the dominant hand and consisted of 60 luminous stimuli. The complex reaction test (CRT) was performed with both hands and also consisted of 60 stimuli. In this last one, the visual stimuli were of two colors, blue or green; to the first, one had to react with the left hand, while to the second, one had to react with the right hand. Two sequences of random numbers were programed for the creation of the tests, one for the SRT and the other for the CRT.

#### Physical Condition

Physical fitness tests were estimated with ALPHA-Fitness Battery (Ruiz et al., 2011) and Eurofit (1993). The following tests were carried out: (a) manual dynamometry, to evaluate the state of the isometric force in the upper train, in both dominant and non-dominant members (the digital dynamometer model TKK-5401 Grip D, Takei, Tokyo, Japan, was used); (b) horizontal jump test, to estimate the force of the lower train; (c) Course–Navette test to analyze the aerobic capacity of the participants, from which the VO2max was indirectly estimated (Léger et al., 1988). For the specific calculation of oxygen consumption, the formula VO2max = 31.025 + 3.238*V* − 3.248*E* + 0.1536*VE* was applied (*V* = the speed reached in the last completed stage; *E* = the age of each participant); (d) speed test 5 × 10 m to analyze travel speed, agility, and general coordination.

#### Selective Attention and Concentration

The D2 attention test was used (Brickenkamp, 2002). Participants had to selectively attend to certain relevant aspects of the task while ignoring irrelevant ones. The test, in this investigation, was administered collectively and lasted between 8 and 10 min. There are 14 lines with 47 elements each (total = 658 items). The elements were letters “d” or “p,” which are accompanied by small lines at the top or bottom of each letter; these small lines could be in pairs or individually. The work that the participant had to do was to check from left to right each line and to mark every letter “d” that is accompanied by two lines (two above, two below, or one above and one below). The participant had 20 s to complete each line. The scores that can be obtained are as follows: TA (total number of attempts), TH (total number of hits), O (omissions or number of relevant stimuli not crossed out), C (omissions or errors), TET [total effectiveness in the test = TP − (O + C)], CON (concentration = TS − C), and VAR [index of variation between the last stimulus analyzed between different rows = (TP+) – (TP−)]. TP+ is the last stimulus analyzed in the row with the most attempted elements, and TP− is the last stimulus analyzed in the row with the fewest attempted elements. This test possesses a test–retest reliability in the original study superior to 0.90.

#### Anthropometry and Body Composition

Anthropometric data were measured with the Tanita body composition analyzer TBF 300 and the mechanical measuring rod kern MSF 200. Data obtained were height, weight, body mass index, and percentage of fat mass.

#### Level of Physical Activity and Manual Dominance

An interview was conducted with each subject whose objective was to collect the extracurricular sports activity of each participant in order to separate the participants according to their level of physical activity. The groups were classified into the following: (1) students who did not engage in any type of extracurricular physical activity; (2) students who engaged in 1–3 h of extracurricular physical activity per week; and (3) students who engaged in more than 3 h of extracurricular physical activity per week. Data were also collected from participants on their dominant hand, i.e., whether they were right-handed or left-handed.

### Procedure

In order to carry out the research, the participating schools were contacted, and permission was requested from the school management for their participation. In addition, informed and written consent was obtained from parents or legal guardians for students to participate. Throughout the research process, the principles established in the Declaration of Helsinki (World Medical Association, 2013) were respected, and approval was obtained from the Ethics Committee of the University of Jaén, Spain (Ref. ABR.16/6).

The tests were performed at the school in 2 days. First, anthropometric data were collected from the participants, and then physical condition tests were performed. Anthropometric measurements were taken in the school gymnasium, with light clothing (shorts and t-shirt), without footwear, and without any metallic object on the body (earrings, chains, watches, etc.). Also, to improve the reliability of body composition measures, the following guidelines were indicated: avoid strenuous exercise the previous day, do not significantly alter the diet the day before the test, wear comfortable clothing, control the taking of medicines that may alter body water levels, and do not retain fluids. As for the physical condition tests, the order was as follows: manual dynamometry, horizontal jump, 5 × 10 m speed test, and 20 m round-trip test. Both were carried out in the school’s sports facilities (multisport courts and gymnasium). The dynamometry, horizontal jump, and speed tests were performed twice, and each participant’s best mark was scored while the Course–Navette test was performed only once.

On the second day, the attention tests and interview were conducted, and the RT was measured. The D2 test was performed collectively in the classroom of the participants. They were previously instructed according to the test manual, and doubts were clarified. The RT was measured in a classroom on an individual basis. First, the simple task was done, and second, the complex task was performed. At the end of the RT test, the student was interviewed to obtain data related to his or her weekly physical activity.

Participants were divided into three groups based on their physical activity habits and routines, not including the physical activity that took place at school during physical education classes. The three groups formed were (a) group 1 (*n* = 57), children who did not carry out any type of physical activity outside school hours; (b) group 2 (*n* = 41), children who carried out between 1 and 3 h a week of physical activity outside school hours; and (c) group 3 (*n* = 21), children who carried out more than 3 h a week of physical activity and/or competed for being federated in some sport.

### Statistical Analysis of Data

The RT measured with the FITLIGHT Trainer was studied. The reliability of the device was studied using the intraclass correlation index (IC), the standard error of measurement (SEM), and the minimal difference (MD). In addition to descriptive data, ANOVAs were performed with the RT as a dependent measure and to see its variation depending on the type of task in all cases, taking into account the position of light in the test, the 10 trials in which the subjects responded, and gender and age.

We analyzed the RT in the three physical activity groups by means of ANOVA of a factor, as well as the Bonferroni and Cohen *d* statistics. Also, correlation analysis (Pearson and Spearman) between physical condition, body composition, and attention measurements with RT values was performed. Linear regression analysis was performed by successive steps to predict the SRT and CRT from the rest of the variables. Data were analyzed with the SPSS statistical program (SPSS Inc., v.20.0, Chicago, IL, United States).

## Results

### Reaction Time (FITLIGHT Trainer)

Table 1 shows the RTs for each of the eight sensor positions and the total mean.

**TABLE 1:** Descriptive statistics (*M* and *SD*) for the RT in the simple and complex tasks of the 119 subjects as a function of the eight positions of the sensor and the total (dominant hand).

		**Total**	**1L**	**2L**	**3L**	**4L**	**5L**	**6L**	**7L**	**8L**
SRT (ms)	M	632.03	613.71	603.19	644.17	713.36	695.51	645.44	572.06	585.38
	SD	97.38	99.21	100.76	124.44	158.51	163.74	123.58	93.093	84.764
CRT (ms)	M	840.68	792.10	840.76	838.69	871.54	854.22	855.60	827.00	862.08
	SD	110.79	129.83	137.2	134.77	152.87	142.43	138.96	132.09	121.21

*SRT, simple reaction time; CRT, complex reaction rime; L, light; M, mean; SD, standard deviation.*

A reliability analysis was performed by calculating the ICC, the SEM, and the MD (Weir, 2005). The ICC_2__,__1_ was calculated for two halves (Wells et al., 2014) by calculating the mean RT for the lights at positions 1, 3, 5, 7 and 2, 4, 6, 8. The type of ICC(2, 1) used considered the effect of trials as a random factor while trials were considered as a sample of possible levels. The results showed ICC_2__,__1_ = 0.92, SEM = 39.87, and MD = 110.51 ms for the simple task (SRT). For the complex task (CRT), the reliability indices were ICC_2__,__1_ = 0.85, SEM = 63.50, and MD = 176.00 ms, which can be considered as adequate reliability indices.

ANOVA of repeated measurements was performed to evaluate the effect of the type of task and the position of light in the RT. The Mauchly sphericity assumption was previously analyzed, obtaining significance for the position (*W* = 0.11; *p* < 0.001) and the interaction of both variables (*W* = *0.48*; *p* < 0.001), so the Greenhouse–Geisser statistic was used. The results showed that the variable type of task was significant (*F*_1__,__118_ = 982.98; *p* < 0.001; η^2^ = 89; 1 − β = 1.00) with lower time in SRT, as well as the position of the light (*F*_3__.__57__,__420__.__92_ = 32.44; *p* < 0.001; η^2^ = 0.22; 1 − β = 1.00) and the interaction between both (*F*_5__.__65__,__668__.__53_ = 23.15; *p* < 0.001; η^2^ = 0.16; 1 − β = 1.00). Peer comparisons for light positions showed significant differences (*p* < 0.05) in all cases except for 1–7, 2–8, 3–6, 3–8, 4–5, and 5–6. The lights located at the ends (1, 2, 7, and 8) obtained lower RT, positions 3 and 6 had intermediate RT, and those located in positions 4 and 5 had higher RT.

In the comparisons by type of task and position of light, it was observed that in the RT of the SRT, there were differences (*p* < 0.05) between all the cases except between 1–2, 1–3, 2–8, 3–6, 4–5, and 7–8. In the CRT, all differences were significant (*p* < 0.001 in all cases except between light 1 and all others, as well as couples 4–7 (*p* = 0.003) and 7–8 (*p* = 0.006). It can be seen that in the SRT, the RT increases for the most central positions, while in the CRT, the position with the least RT is position 1, followed by position 7, with the rest having more or less the same times, the greater being that relating to light in position 4.

An analysis of the RT was done according to the test in which the RT was measured with the aim of studying whether fatigue affects the RT differentially. We have considered the average RT for every 10 trials in each subject. Since each of us performed 60 trials, we compared six dozen trials (Table 2).

**TABLE 2:** Descriptive statistics (*M* and *SD*) for the RT in the simple and complex tasks of the 119 subjects according to the six dozen trials.

		**1D**	**2D**	**3D**	**4D**	**5D**	**6D**
SRT (ms)	M	639.84	612.04	647.21	621.99	643.99	627.12
	SD	107.79	100.49	111.90	107.79	116.13	110.20
CRT (ms)	M	768.00	833.64	848.97	859.74	868.21	865.53
	SD	118.51	132.04	121.01	141.32	138.99	139.93

*SRT, simple reaction time; CRT, complex reaction time; D, dozen; M, mean; SD, standard deviation.*

An ANOVA of repeated measurements was performed to study the effect of the type of task and tests in RT, with the Mauchly sphericity test previously performed, being significant for the variable trial (*W* = 0.74; *p* = 0.001) but not for the interaction type of task × trial (*W* = 0.83; *p* = 0.079). Therefore, only Greenhouse–Geisser was applied in the first case. The variable type of task, as it happened before, was significant (*F*_1__,__118_ = 10,008.22; *p* < 0.001; η^2^ = 0.89; 1 − β = 1.00), with time being higher in the complex task. In terms of the trial, significant differences were obtained between the six levels (*F*_4__.__42__,__521__.__69_ = 19.08; *p* < 0.001; η^2^ = 0.14; 1 − β = 1.00) and also in the interaction of type of task × trial (*F*_5__,__590_ = 22.96; *p* < 0.001; η^2^ = 0.16; 1 − β = 1.00). In the comparisons of Bonferroni by pairs of the trial variable, there was a significance (*p* < 0.05) of the first and second tens with all the others, while there were no differences between the third, fourth, fifth, and sixth tens. That is to say, the RT was lower in the first ten, increased significantly in the second, increased significantly in the third, and did not increase until the end.

As for the interaction in the simple type, differences were found between tens 1–3, 2–3, 2–5, 3–4, and 4–5 (*p* < 0.01) although there is no tendency to increase or decrease as the tens increase, but rather a sawtooth trend was produced, the RT being greater in the odd tens than in the pairs. However, in the complex task, there was an increase in the RT as the tens increased except between the fifth and sixth tens that had practically equal RT although only these increases are significant between the first ten compared with all the others (*p* < 0.01) and the second ten compared with the fifth (*p* < 0.01) and sixth (*p* = 0.04) tens.

Finally, with regard to the FITLIGHT system, the aim was to study whether there were differences between the RTs according to the two tasks in terms of gender and age (Table 3).

**TABLE 3:** Descriptive statistics (*M* and *SD*) for the RT in the simple and complex tasks according to gender and age.

	**Age**	**10**		**11**		**12**	
		
	**Gender**	**Boys**	**Girls**	**Boys**	**Girls**	**Boys**	**Girls**
SRT (ms)	M	642.58	668.22	591.15	632.62	551.51	625.00
	SD	116.58	73.23	75.91	115.75	55.76	86.66
CRT (ms)	M	874.58	868.81	800.74	832.97	767.80	816.83
	SD	131.42	94.51	93.13	123.77	53.80	99.88

*SRT, simple reaction time; CRT, complex reaction time; M, mean; SD, standard deviation.*

ANOVA showed significant differences for the variable type of task (*F*_1__,__113_ = 822.97; *p* < 0.001; η^2^ = 0.88; 1 − β = 1.00) and age (*F*_2_,_113_ = 5.86; *p* = *0.004*; η^2^ = 0.09; 1 − β = 0.87) but not gender (*F*_1_,_113_ = 3.56; *p* = 0.06; η^2^ = 0.03; 1 − β = 0.46). There was also no significance in the interaction type of task × age (*F*_2__,__113_ = 0.38; *p* = 0.68; η^2^ = 0.01; 1 − β = 0.11), type of task × gender (*F*_1__,__113_ = 2.23; *p* = 0.14; η^2^ = 0.02; 1 − β = 0.32), age × gender (*F*_2__,__112_ = 0.63; *p* = 0.54; η^2^ = 0.01; 1 − β = 0.15), or type of task × age × gender (*F*_2__,__113_ = 0.27; *p* = 0.76; η^2^ = 0.01; 1 − β = 0.09).

### Reaction Time and Physical Activity

Table 4 shows descriptive and normal analyses (Kolmogorov–Smirnov, *n* > 50; Shapiro–Wilk, *n* < 50) of reaction time (simple and complex) for each physical activity group.

**TABLE 4:** Descriptive statistics (*M* and *SD*) for the RT in the simple and complex tasks according to physical activity.

	***M***	***SD***	***S***	***K***	**K–S**	**S–W**
SRT group 1 (ms)	646.70	108.41	0.98	1.66	0.57	–
SRT group 2 (ms)	635.17	81.02	–0.24	–0.53	–	0.98
SRT group 3 (ms)	577.94	76.98	0.51	–0.12	–	0.92
CRT group 1 (ms)	847.12	123.83	0.82	1.03	0.30	–
CRT group 2 (ms)	865.08	95.32	–0.29	0.76	–	0.97
CRT group 3 (ms)	775.54	74.30	0.16	–0.58	–	0.96

*SRT, simple reaction time; CRT, complex reaction time; M, mean; SD, standard deviation; S, skewness; K, kurtosis; K–S, Kolmogorov–Smirnov; S–W, Shapiro–Wilk.*

**FIGURE 1:**
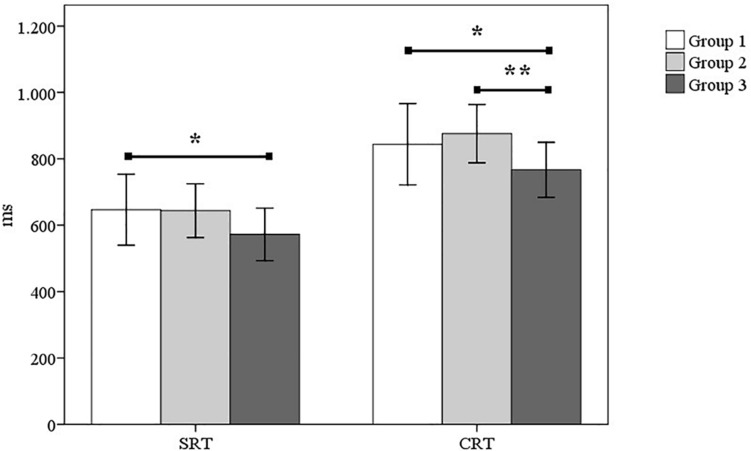
Differences between groups for SRT and CRT. SRT, simple reaction time; CRT, complex reaction time. ^∗^*p* < 0.05; ^∗∗^*p* < 0.01.

ANOVA was performed for each RT measure, with differences observed between groups for SRT (*F*_2_*_,_*_116_ = 4.43; *p* < 0.05) and CRT (*F*_2__,__116_ = 5.04; *p* < 0.01). Bonferroni’s statistic was applied to analyze the differences between the groups, observing differences between group 3 and group 1 in SRT (*p* < 0.05, *d* = −0.68, 95% CI [−1.19, −0.17]) and CRT (*p* < 0.05, *d* = −0.63, 95% CI [−1.14, −0.12]), as well as differences between group 3 and group 2 in CRT (*p* < 0.01, *d* = −1.01, 95% CI [−1.56, −0.45]) (Figure 1).

### Reaction Time, Physical Condition, and Attention

Table 5 shows the descriptive statistics for the variables of physical condition, selective attention, and RT, as well as the existing correlation with the RT in the two tasks.

**TABLE 5:** Descriptive statistics for the variables of physical condition, selective attention, and anthropometry and their correlation with the two RT tasks.

	***M***	***SD***	***S***	***K***	**K–S**	**TS**	**TC**
BMI	19.96	3.27	0.76	0.89	0.92	–0.05	–0.01
% fat mass	22.73	8.37	0.28	–0.13	0.68	–0.02	–0.01
Dyn_dom	16.49	3.64	0.25	–0.76	0.79	–0.28^∗∗^	–0.36^∗∗∗^
Dyn_non-dom	15.64	3.99	0.68	0.61	1.09	–0.29^∗∗^	–0.32^∗∗^
HJT	121.69	22.85	0.05	0.10	0.54	–0.31^∗∗^	–0.25^∗∗^
5 × 10	22.60	2.05	0.75	0.93	1.27	0.39^∗∗∗^	0.21^∗^
VO2max	44.16	4.26	0.34	–0.05	1.25	–0.27^∗∗^	–0.26^∗∗^
D2_TA	329.34	66.67	0.85	3.11	0.77	–0.09	–0.10
D2_TH	125.73	24.65	–0.47	0.31	0.63	−0.22^∗^	–0.23^∗∗^
D2_O	9.95	12.96	2.34	7.70	1.79^∗∗^	–0.04	–0.05
D2_C	6.12	9.85	2.93	10.33	2.59^∗∗∗^	0.01	0.10
D2_TET	307.71	56.13	–0.38	0.39	0.65	−0.19^∗^	–0.25^∗∗^
D2_CON	118.42	32.41	–0.82	1.30	0.89	–0.24^∗∗^	–0.31^∗∗^
D2_VAR	15.57	7.65	1.33	2.04	1.84^∗∗^	–0.13	–0.06
SRT	632.03	97.38	0.77	1.55	0.63	–	–
CRT	840.68	110.79	0.59	0.95	0.92	–	–

*M, mean; SD, standard deviation; S, skewness; K, kurtosis; K–S, Kolmogorov–Smirnov; BMI, body mass index; Dyn_dom, dynamometry (dominant); Dyn_non-dom, dynamometry (non-dominant); HJT, horizontal jump test (cm); 5 × 10, speed test 5 × 10 m (s); VO2max, maximum oxygen consumption (ml/kg/min); D, D2 test; TA, total number of attempts; TH, total number of hits; O, omissions; C, commissions; TET, total effectiveness in the test; CON, concentration index; VAR, variation index; SRT, simple reaction time; CRT, complex reaction time. ^∗^*p* < 0.05; ^∗∗^*p* < 0.01; ^∗∗∗^*p* < 0.001.*

Two regression analyses (successive steps) were performed, one for SRT and another for CRT, using as predictive variables the physical condition and measurements of the D2 attention test. The linearity assumptions were met in the relationship between predictor variables and criteria, homoscedasticity, and normal waste distribution. Durbin–Watson values were 2.05 and 1.93, so it can be assumed that the waste is independent, and the assumption of independence of the independent variables with respect to the dependent one is fulfilled (Pardo and Ruiz, 2005).

In the case of SRT, the regression model included two variables, velocity test (β = 0.30) and dynamometry (non-dominant) (β = −0.23). The following values were obtained for this model: *R* = 0.43; *R*^2^ = 0.19; *R*^2^ adjusted = 0.17; *F* = 11.09; *p* < 0.001. The tolerance (0.90) and variance inflation factor (1.11) values of the model were adequate.

In the case of CRT, the regression model included dynamometry (dominant) (β = −0.40), concentration (D2-CON) (β = −0.40), and VO2max (β = −0.40). The following values were obtained for this model: *R* = 0.44; *R*^2^ = 0.20; *R*^2^ adjusted = 0.17; *F* = 7.92; *p* < 0.001. The tolerance (0.93–0.97) and variance inflation factor (1.04–1.08) values of the model were adequate.

## Discussion

The objective of the present study was to analyze the relationships between RT with selective attention and concentration and also with weekly physical activity volume and physical fitness in a sample of children with ages from 10 to 12 years. Likewise, this investigation evaluated whether cognitive functioning and physical condition were adequate predictors of RT, both simple and complex. The results showed the relationship between RT and weekly physical activity volume, physical fitness, selective attention, and concentration. In general, physical fitness predicted RT scores. However, only cognitive functioning was a predictor of complex RT.

First, the amount of weekly physical activity has been related to simple and complex RTs. Those who did more hours of physical activity a week showed less RT on both tasks. These results are congruent with previous research that had pointed out these associations (Zwierko et al., 2014; Jain et al., 2015; Okubo et al., 2017; van de Water et al., 2017). Although the groups have not been divided according to the type of physical activity or sports performed, these results would support the idea that physical practice could be a useful activity to develop RT. When doing physical exercise, it is necessary to act effectively in a series of events, so this type of practice could have favored an increase in the capacity to act with greater speed and effectiveness in similar tasks (Nuri et al., 2013; van de Water et al., 2017; Lynall et al., 2018; Walton et al., 2018), transferring this ability to others such as those evaluated in this work (Rabiner et al., 2010; Kirk et al., 2017).

Second, relations have been observed between the level of physical fitness, attentional capacity, and concentration with RT, which would approximate studies that had previously pointed out this phenomenon (Vaportzis et al., 2013; Jehu et al., 2015; Luque-Casado et al., 2016; Westfall et al., 2018). Being a correlational study, it is not possible to determine causal effects, but according to the findings found in various investigations, there could be multiple links between the variables studied. Reloba-Martínez et al. (2017) highlighted that a high-intensity exercise program had positive effects on selective attention and fitness. Therefore, the combination of physical exercise and the development of cognitive functioning could be an appropriate formula to improve RT in people.

Specifically, linear regression analyses have shown that the simple RT has been predicted solely by physical condition measurements. However, the model generated for the complex RT has combined physical condition and attentional measures. Specifically, the dominant manual dynamometry, concentration, and maximum oxygen consumption have been included variables. This is consistent with previous studies that had highlighted a greater relationship between these measures in situations requiring greater cognitive control (Westfall et al., 2018). The complex RT requires selecting a response to different possibilities (Boisgontier et al., 2014), so the demands to respond effectively are greater. In this work, the RT has been evaluated by means of an oculo-manual coordination task using the FITLIGHT Trainer system, which suggests that a better physical condition and a greater capacity to concentrate could have influenced the developed behavior, as indicated by the data obtained.

It is interesting to note that the physical fitness measurements that predicted the values in complex RT were manual dynamometry and maximum oxygen consumption. Cardiorespiratory fitness has been widely documented as an ability linked to improved cognitive ability and improved performance on tasks requiring cognitive control (Kao et al., 2017; Westfall et al., 2018). However, the dominant manual dynamometry has been the strongest factor in the regression equation. This could have happened because of the type of task analyzed, which required a quick and efficient motor action of the upper limbs in the face of the visual stimuli of the FITLIGHT Trainer test. Probably, the neuromuscular requirements intrinsic to the task itself could have conditioned the results found. This could indicate that it is important, when carrying out this type of studies, to take into account the type of activity analyzed, given that the nature of the activity could modulate the conclusions derived from it.

This paper presents a number of limitations. On the one hand, the analysis of oxygen consumption has been carried out indirectly, which is data with a certain margin of error. In future works, it would be interesting to use a type of direct gas analysis test in an incremental stress test to obtain more reliable data. On the other hand, the type of design used does not allow establishing causal relationships between the variables analyzed. It would be interesting to carry out longitudinal or quasi-experimental work to observe how the data evolve as a function of changes in the physical condition or in the cognitive functioning of the study sample. In any case, this research carries out an interesting analysis in which it has linked variables of cognitive functioning, physical practice, and physical condition with RT, providing data that allow us to delve deeper into this phenomenon and that increase empirical evidence of the internal factors that could condition RT in preadolescents.

The findings found in this study suggest that better development of attention and concentration, as well as physical condition, could help improve RT at these ages. This could contribute to improving efficiency in tasks that are important for the personal and social growth of children and adolescents. Therefore, it would be interesting to contribute to its improvement when considering psychomotor development programs in this population.

## Data Availability Statement

The datasets generated for this study are available on request to the corresponding author.

## Ethics Statement

The studies involving human participants were reviewed and approved by Ethics Committee of the University of Jaén, Spain (Ref. ABR.16/6). Written informed consent to participate in this study was provided by the participants’ legal guardian/next of kin.

## Author Contributions

SB, IM, AH-M, VM-S, RR, and RJ-R participated in the study design and data collection, performed the statistical analyses, contributed to the interpretation of the results, wrote the manuscript, and approved the final manuscript. RR, AH-M, SB, and IM conceived the study and participated in its design and coordination. AH-M, VM-S, RR, RJ-R, SB, and IM contributed to the interpretation of the results, and reviewed and provided feedback to the manuscript. All authors made substantial contributions to the final manuscript.

## Conflict of Interest

The authors declare that the research was conducted in the absence of any commercial or financial relationships that could be construed as a potential conflict of interest.

## References

[B1] Adleman N. E. Chen G. Reynolds R. C. Frackman A. Razdan V. Weissman D. H. (2016). Age-related differences in the neural correlates of trial-to-trial variations of reaction time. *Dev. Cogn. Neurosci.* 19 248–257. 10.1016/j.dcn.2016.05.001 27239972 PMC5099497

[B2] Appelbaum L. G. Erickson G. (2018). Sports vision training: a review of the state-of-the-art in digital training techniques. *Int. Rev. Sport Exerc. Psychol.* 11 160–189. 10.1080/1750984X.2016.1266376

[B3] Baayen R. H. Milin P. (2010). Analyzing reaction times. *Int. J. Psychol. Res.* 3 12–28. 10.21500/20112084.807

[B4] Boisgontier M. P. Wittenberg G. F. Fujiyama H. Levin O. Swinnen S. P. (2014). Complexity of central processing in simple and choice multilimb reaction-time tasks. *PLoS One* 9:e90457. 10.1371/journal.pone.0090457 24587371 PMC3938735

[B5] Brickenkamp R. (2002). *D2, Test de Atención.* Madrid: TEA Ediciones.

[B6] Chun M. M. Golomb J. D. Turk-Browne N. B. (2011). A taxonomy of external and internal attention. *Annu. Rev. Psychol.* 62 73–101. 10.1146/annurev.psych.093008.100427 19575619

[B7] Deary I. J. Der G. (2005). Reaction time, age, and cognitive ability: longitudinal findings from age 16 to 63 years in representative population samples. *Aging Neuropsychol. Cogn.* 12 187–215. 10.1080/13825580590969235

[B8] Der G. Deary I. J. (2006). Age and sex differences in reaction time in adulthood: results from the United Kingdom health lifestyle survey. *Psychol. Aging* 21 62–73. 10.1037/a0015515 16594792

[B9] Eurofit (1993). *Eurofit Tests of Physical Fitness*, 2nd Edn, Strasbourg: Committee of Experts on Sports Research.

[B10] Fischer M. V. Stone J. Hawkes T. D. Eveland E. Strang A. J. (2015). Integrative physical and cognitive training development to better meet airmen mission requirements. *Procedia Manuf.* 3 1580–1586. 10.1016/j.promfg.2015.07.445

[B11] Gentier I. Augustijn M. Deforche B. Tanghe A. De Bourdeaudhuij I. Lenoir M. (2013). A comparative study of performance in simple and choice reaction time tasks between obese and healthy-weight children. *Res. Dev. Disabil.* 34 2635–2641. 10.1016/j.ridd.2013.04.016 23751303

[B12] Giuliano R. J. Karns C. M. Neville H. J. Hillyard S. A. (2014). Early auditory evoked potential is modulated by selective attention and related to individual differences in visual working memory capacity. *J. Cogn. Neurosci.* 26 2682–2690. 10.1162/jocn-a-00684 25000526 PMC4327887

[B13] Gomez-Ramirez M. Hysaj K. Niebur E. (2016). Neural mechanisms of selective attention in the somatosensory system. *J. Neurophysiol.* 116 1218–1231. 10.1152/jn.00637.2015 27334956 PMC5018055

[B14] Greenhouse I. King M. Noah S. Maddock R. J. Ivry R. B. (2017). Individual differences in resting corticospinal excitability are correlated with reaction time and GABA content in motor cortex. *J. Neurosci.* 37 2686–2696. 10.1523/JNEUROSCI.3129-16.2017 28179557 PMC5354322

[B15] Greimel E. Wanderer S. Rothenberger A. Herpertz-Dahlmann B. Konrad K. Roessner V. (2011). Attentional performance in children and adolescents with tic disorder and co-occurring attention-deficit/hyperactivity disorder: new insights from a 2x2 factorial design study. *J. Abnorm. Child Psych.* 39 819–828. 10.1007/s10802-011-9493-7 21331638 PMC3111554

[B16] Hillman C. H. Pontifex M. B. Raine L. B. Castelli D. M. Hall E. E. Kramer A. F. (2009). The effect of acute treadmill walking on cognitive control and academic achievement in preadolescent children. *Neuroscience* 159 1044–1054. 10.1016/j.neuroscience.2009.01.057 19356688 PMC2667807

[B17] Jain A. Bansal R. Kumar A. Singh K. D. (2015). A comparative study of visual and auditory reaction times on the basis of gender and physical activity levels of medical first year students. *Int. J. Appl. Basic Med. Res.* 5 124–127. 10.4103/2229-516X.157168 26097821 PMC4456887

[B18] Jayaswal A. A. (2016). Comparison between auditory and visual simple reaction times and its relationship with gender in 1st year MBBS students of jawaharlal nehru medical college, Bhagalpur, Bihar. *Int. J. Med. Res. Rev.* 4 1228–1232. 10.17511/ijmrr.2016.i07.26

[B19] Jehu D. A. Desponts A. Paquet N. Lajoie Y. (2015). Prioritizing attention on a reaction time task improves postural control and reaction time. *Int. J. Neurosci.* 125 100–106. 10.3109/00207454.2014.907573 24655152

[B20] Jensen A. (2006). *Cloking the Mind: Mental Chronometry and Individual Differences.* Amsterdam: Elsevier.

[B21] Kao S. C. Westfall D. R. Soneson J. Gurd B. Hillman C. H. (2017). Comparison of the acute effects of high-intensity interval training and continuous aerobic walking on inhibitory control. *Psychophysiology* 54 1335–1345. 10.1111/psyp.12889 28480961

[B22] Kirk H. Gray K. Ellis K. Taffe J. Cornish K. (2017). Impact of attention training on academic achievement, executive functioning, and behavior: a Randomized controlled trial. *Am. J. Intellect.* 122 97–117. 10.1352/1944-7558-122.2.97 28257246

[B23] Kuang S. (2017). Is reaction time an index of white matter connectivity during training? *Cogn. Neurosci.* 8 126–128. 10.1080/17588928.2016.1205575 27472472

[B24] Leckie R. L. Oberlin L. E. Voss M. W. Prakash R. S. Szabo-Reed A. Chaddock-Heyman L. (2014). BDNF mediates improvements in executive function following a 1-year exercise intervention. *Front. Hum. Neurosci.* 8:985. 10.3389/fnhum.2014.00985 25566019 PMC4263078

[B25] Léger L. A. Mercier D. Gadoury C. Lambert J. (1988). The multistage 20 metre shuttle run test for aerobic fitness. *J. Sport Sci.* 6 93–101. 10.1080/02640418808729800 3184250

[B26] Luque-Casado A. Perakakis P. Hillman C. H. Kao S. C. Llorens F. Guerra P. (2016). Differences in sustained attention capacity as a function of aerobic fitness. *Med. Sci. Sports Exerc.* 48 887–895. 10.1249/MSS.0000000000000857 26694844

[B27] Lynall R. C. Blackburn J. T. Guskiewicz K. M. Marshall S. W. Plummer P. Mihalik J. P. (2018). Reaction time and joint kinematics during functional movement in recently concussed individuals. *Arch. Phys. Med. Rehabil.* 99 880–886. 10.1016/j.apmr.2017.12.011 29337022

[B28] Metin B. Wiersema J. R. Verguts T. Gasthuys R. van Der Meere J. J. Roeyers H. (2016). Event rate and reaction time performance in ADHD: testing predictions from the state regulation deficit hypothesis using an ex-Gaussian model. *Child Neuropsychol.* 22 99–109. 10.1080/09297049.2014.986082 26835532

[B29] Mudric M. Cuk I. Nedeljkovic A. Jovanovic S. Jaric S. (2015). Evaluation of Video-based method for the measurement of reaction time in specific sport situation. *Int. J. Perf. Anal. Sports* 15 1077–1089. 10.1080/24748668.2015.11868852

[B30] Nuri L. Shadmehr A. Ghotbi N. Attarbashi Moghadam B. (2013). Reaction time and anticipatory skill of athletes in open and closed skill-dominated sport. *Eur. J. Sport Sci.* 13 431–436. 10.1080/17461391.2012.738712 24050458

[B31] Okubo Y. Schoene D. Lord S. R. (2017). Step training improves reaction time, gait and balance and reduces falls in older people: a systematic review and meta-analysis. *Br. J. Sports Med.* 51 586–593. 10.1136/bjsports-2015-095452 26746905

[B32] Pardo A. Ruiz M. A. (2005). *Data Analysis with SPSS 13 Base.* Madrid: McGraw Hill.

[B33] Petersen S. E. Posner M. I. (2012). The attention system of the human brain: 20 years after. *Annu. Rev. Neurosci.* 35 73–89. 10.1146/annurev-neuro-062111-150525 22524787 PMC3413263

[B34] Prinzmetal W. McCool C. Park S. (2005). Attention: reaction time and accuracy reveal different mechanisms. *J. Exp. Psychol. Gen.* 134 73–92. 10.1037/0096-3445.134.1.73 15702964

[B35] Rabiner D. L. Murray D. W. Skinner A. T. Y. Malone P. S. (2010). A randomized trial of two promising computer-based interventions for students with attention difficulties. *J. Abnorm. Child Psych.* 38 131–142. 10.1007/s10802-009-9353-x 19697119

[B36] Reloba-Martínez S. Reigal R. E. Hernández-Mendo A. Martínez-López E. J. Martín-Tamayo I. Chirosa-Ríos L. J. (2017). Effects of vigorous extracurricular physical exercise on the attention of schoolchildren. *Rev. Psicol. Deporte.* 26 29–36.

[B37] Ruiz J. R. Castro-Piñero J. España-Romero V. Artero E. G. Ortega F. B. Cuenca M. M. (2011). Field-based fitness assessment in young people: the ALPHA health-related fitness test battery for children and adolescents. *Br. J. Sport Med.* 45 518–524. 10.1136/bjsm.2010.075341 20961915

[B38] Ruschel C. Haupenthal A. Hubert M. Fontana H. B. Pereira S. M. Roesler H. (2011). Simple reaction time in soccer players from differing categories and field positions. *Motricidad* 7 73–82.

[B39] Sant’Ana J. Franchini E. da Silva V. Diefenthaeler F. (2016). Effect of fatigue on reaction time, response time, performance time, and kick impact in taekwondo roundhouse kick. *Sport Biomech.* 16 201–209. 10.1080/14763141.2016.1217347 27592682

[B40] Syväoja H. J. Tammelin T. H. Ahonen T. Kankaanpää A. Kantomaa M. T. (2014). The associations of objectively measured physical activity and sedentary time with cognitive functions in school-aged children. *PLoS One* 9:e103559. 10.1371/journal.pone.0103559 25061820 PMC4111611

[B41] Tamm L. Epstein J. N. Peugh J. L. Nakonezny P. A. Hughes C. W. (2013). Preliminary data suggesting the efficacy of attention training for school-aged children with ADHD. *Dev. Cogn. Neurosci.* 4 16–28. 10.1016/j.dcn.2012.11.004 23219490 PMC3617931

[B42] Tønnessen E. Haugen T. Shalfawi S. A. (2013). Reaction time aspects of elite sprinters in athletic world championships. *J. Strength Cond. Res.* 27 885–892. 10.1519/JSC.0b013e31826520c3 22739331

[B43] van de Water T. Huijgen B. Faber I. Elferink-Gemser M. (2017). Assessing cognitive performance in badminton players: a reproducibility and validity study. *J. Hum. Kinet.* 55 149–159. 10.1515/hukin-2017-0014 28210347 PMC5304283

[B44] Vaportzis E. Georgiou-Karistianis N. Stout J. C. (2013). Dual task performance in normal aging: a comparison of choice reaction time tasks. *PLoS One* 8:e60265. 10.1371/journal.pone.0060265 23555937 PMC3605385

[B45] Vences de Brito A. Salva C. Cid L. Ferreira R. Marques A. (2011). Attention and reaction time in shotokan karate practitioners. *J. Asian Martial Arts* 1 141–156.

[B46] Walton C. C. Keegan R. J. Martin M. Hallock H. (2018). The potential role for cognitive training in sport: more research needed. *Front. Psychol.* 9:1121. 10.3389/fpsyg.2018.01121 30018585 PMC6037849

[B47] Weir J. P. (2005). Quantifying test-retest reliability using the intraclass correlation coefficient and the SEM. *J. Strength Cond. Res.* 19 231–240. 10.1519/15184.1 15705040

[B48] Wells A. J. Hoffman J. R. Beyer K. S. Jajtner A. R. González A. M. Townsend J. R. (2014). Reliability of the dynavision^TM^ d2 for assessing reaction time performance. *J. Sport Sci. Med.* 13 145–150. 24570618 PMC3918550

[B49] Westfall D. R. Gejl A. K. Tarp J. Wedderkopp N. Kramer A. F. Hillman C. H. (2018). Associations between aerobic fitness and cognitive control in adolescents. *Front. Psychol.* 9:1298. 10.3389/fpsyg.2018.01298 30158882 PMC6104451

[B50] Woods D. L. Wyma J. M. Yund E. W. Herron T. J. Reed B. (2015). Factors influencing the latency of simple reaction time. *Front. Hum. Neurosci.* 9:131. 10.3389/fnhum.2015.00131 25859198 PMC4374455

[B51] World Medical Association (2013). World Medical Association Declaration of Helsinki: ethical principles for medical research involving human subjects. *JAMA* 310 2191–2194. 10.1001/jama.2013.281053 24141714

[B52] Zurek M. Cosmi S. Cicchela A. Roi G. S. (2015). “Simple and complex reaction time at visual stimulation, before and after a rehabilitation after knee surgery in football players,” in *Poster Presented in XXIV International Conference on Sports Rehabilitation and Traumatology*, London.

[B53] Zwierko T. Florkiewicz B. Slawomir F. Kszak-Krzyzanowska A. (2014). The ability to maintain atention during visuomotor task performance in handball players and non athletes. *Cent. Eur. J. Sport Sci. Med.* 7 99–106.

